# Systemic Evaluation of Administered FITC-Dextran or Evans Blue Dye to Determine Intestinal Barrier Permeability in Nonhuman Primates

**DOI:** 10.20411/pai.v11i2.1054

**Published:** 2026-08-25

**Authors:** Elizabeth G. Bodykevich, Carol L. Vinton, Alexandra M. Ortiz, Jason M. Brenchley

**Affiliations:** 1 Barrier Immunity Section, Laboratory of Viral Diseases, Division of Intramural Research, National Institute of Allergy and Infectious Diseases (NIAID), National Institutes of Health (NIH), Bethesda, Maryland

**Keywords:** SIV, HIV, Microbial Translocation, Evans Blue, Uracil

## Abstract

**Background:**

Microbial translocation is a key contributor to chronic immune activation during HIV infection. Current methods of measuring microbial translocation are limited in sensitivity and are largely indirect, underscoring the need for more precise and direct techniques that can be followed longitudinally. We aimed to address this gap by applying modalities commonly employed in mouse models of intestinal permeability.

**Methods:**

We explored whether oral FITC-Dextran or suppository-delivered Evans blue dye in nonhuman primates could be leveraged to approximate intestinal barrier disruption in healthy and SIV-infected rhesus macaques.

**Results:**

After administration, FITC-Dextran was detected at comparable concentrations in the plasma of healthy and chronically infected animals, whereas healthy animals had greater concentrations of Evans blue dye as compared to chronically infected animals. Evans blue was reliably detected in phagocytic cells across tissue types, with detection often highest in the rectum, liver, and spleen biopsies. Exogenous uracil modulated an increase in intestinal barrier permeability observed through standard markers, which was not reliably reflected on Evans blue uptake.

**Conclusion:**

These data demonstrate that dye-based measurements of intestinal permeability routinely used in murine models are not sufficient in nonhuman primates, and that common biomarkers and/or sugar permeability and immunohistochemical approaches are still preferred.

## INTRODUCTION

Maintenance of the structure and function of the intestinal epithelium is central to human health. The gastrointestinal (GI) tract is home to trillions of microbes comprising the gut microbiome [1]. Keeping these microbes within the lumen is critical to maintaining host health [2]. In diseases such as colorectal cancer, inflammatory bowel disease, cirrhosis, and human immunodeficiency virus (HIV) infection, loss of barrier function and increased intestinal permeability result in microbial translocation: the movement of microbes and/or their products from the gut lumen into systemic circulation [3–7]. Microbial translocation is a key contributor to chronic immune activation, which can advance disease progression and worsen health outcomes [7]. In people living with HIV, use of antiretroviral therapies (ART) successfully increases life span and reduces viral replication; however, chronic immune activation and mucosal dysfunction persist [8–10]. Further, individuals who initiate ART during chronic HIV infection are at increased risk for non-HIV-associated co-morbidities including cancers and cardiovascular disease [8]. Due to the significant implications of microbial translocation in disease pathogenesis, it is important to have reliable methods for its measurement.

Reliable methods for directly measuring microbial translocation are scant. Concentrations of LPS in plasma can be measured directly using a Limulus Amebocyte Lysate (LAL) assay [5, 11, 12]. Limitations of the LAL assay include a high risk of contamination, dependence on a limited supply chain, and underestimating the degree of translocation due to inhibitory effects from plasma components [7, 11, 13, 14]. Furthermore, this method only provides information about Gram-negative bacteria, without producing identities of translocating bacteria. Thus qPCR and/or sequencing of bacterial DNA isolated from plasma is preferred [12, 15, 16]. The conserved and hypervariable regions of the 16S rRNA gene allow for the distinction of bacterial genus and provide information on relative abundance [17]. While 16S rRNA sequencing and/or qPCR is informative, it is also expensive, highly susceptible to contamination within the enzymes used for measurement, and limited by the amount and stability of circulating bacterial DNA [7].

Alternatively, indirect measures of microbial translocation involve plasmatic measurements of biomarkers made in response to microbial product-mediated stimulation, including soluble CD14 (sCD14), LPS binding protein, and endotoxin core antibodies (EndoCAb) [5, 7, 18, 19]. Enzyme-linked immunosorbent assays (ELISAs) for these biomarkers are generally reliable and reproducible; however, these markers are also produced *in vivo* in response to stimulants beyond microbial products, and none is singularly accepted as the benchmark [20, 21]. Intestinal fatty acid binding protein 2 (iFABP2) and zonulin are common plasma biomarkers for intestinal permeability that are strong predictors of mortality in treated HIV infection, although these measurements don’t always correlate with sCD14 measurements [20, 22, 23]. Furthermore, although zonulin was originally identified as pre-haptoglobin-2, the term has since been expanded to include a family of structurally related proteins, and commercially available zonulin assays may not specifically detect pre-haptoglobin-2 [24, 25]. Permeability of the small intestine is commonly measured by the ratio of orally ingested lactulose to mannitol detected in urine excretion, although interference from high sugar diets and colonic bacterial degradation of the sugars does not make it an ideal method for measuring colonic or whole gut permeability [21, 26, 27]. Thus, non-invasive and reliable biomarkers are needed to determine permeability of the GI tract.

In murine models of GI tract damage, labeled tracers, such as FITC-Dextran, can be orally administered and measured within the plasma as a surrogate of intestinal permeability by fluorescence [28–30]. Alternatively, non-cell permeable Evans blue — an azo dye with high affinity for serum albumin — has been used to assess blood-brain barrier permeability as well as vascular permeability in rodents [31, 32]. Here, we sought to determine if these modalities may provide a reasonable approach to measure intestinal permeability in nonhuman primates. Healthy and SIV-infected rhesus macaques were treated with FITC-Dextran by oral gavage or Evans blue dye dissolved in cocoa butter as a rectal suppository to assess intestinal permeability at various stages of infection. The results from our study may provide insight into important improvements needed to accurately measure intestinal barrier integrity and microbial translocation.

## METHODS

### Animals and Treatments

Nineteen adult rhesus macaques were assessed for microbial translocation (Supplementary Table 1). At the beginning of each study, animals were either healthy (SIV-) or chronically infected with SIVmac239X. One animal was infected during the study with 1,000 TCID_50_ SIVmac239X intravenously. Infection was confirmed by plasma viral loads (Supplementary Table 1).

FITC-Dextran (4 kDa, CAS#60842-46-8, Sigma-Aldrich #46944) was dissolved in phosphate-buffered saline (PBS) (100 mg/mL) the morning of treatment and was sterile filtered before administering it (500 mg/kg) once to 6 healthy, and 6 SIV-infected macaques under sedation via oral gavage. The evening prior to treatment, no earlier than 2:30 pm, water was removed from cages to normalize plasma viscosity and allow for even adsorption of FITC-Dextran. The morning of treatment, before 7:30 am, water was returned to cages and animals were weighed and assessed for hydration levels during sedation. No animals were found to be dehydrated by skin tent.

Evans Blue dye (ThermoFisher Scientific #A16774.09) was dissolved (0.075% w/w) in 15 g of cocoa butter (MP Biomedicals #905417) at 37°C. Individual aliquots were stored at -20°C and thawed in a 37°C water bath before being administered as a colonic suppository to anesthetized animals, 3 times per week. As a control, a singular animal received an enema with Normosol-R pH 7.4 (1 ml/kg, ICU Medical #796709) 3 times per week as previously described [33].

Uracil (MP Biomedicals #103204) was administered orally (N = 2) or as a colonic suppository (N = 3) to healthy and SIV-infected macaques. Oral uracil (125 mg/m^2^) was delivered twice daily, sprinkled in meals. Suppository uracil (1500 mg/dose) was delivered in a cocoa butter suppository (10% w/w) containing 0.075% Evans Blue thrice weekly by video-guided endoscopy in anesthetized animals.

The National Institute of Allergy and Infectious Diseases (NIAID) Division of Intramural Research Animal Care and Use Program, as part of the National Institutes of Health (NIH) Intramural Research Program, approved all the experimental procedures (protocol LVD26E). The Program complies with all applicable provisions of the Animal Welfare Act and other federal statutes and regulations relating to animals. Animals were housed and cared for at the NIH Animal Center, under the supervision of the Association for the Assessment and Accreditation of Laboratory Animal Care (AAALAC)-accredited Division of Veterinary Resources and as recommended by the Office of Animal Care and Use Nonhuman Primate Management Plan. Husbandry and care met the standards set forth by the Animal Welfare Act, Animal Welfare Regulations, as well as The Guide for the Care and Use of Laboratory Animals (8th Edition). The physical conditions of the animals were monitored daily.

### Sample Collection

Samples were collected and processed from anesthetized animals as previously described [33]. In brief, blood and tissue biopsies were collected at predetermined timepoints throughout the studies. Animals were fasted overnight and on the morning of surgery were sedated with Telazol (3–4mg/kg, IM) and further anesthetized with isoflurane gas by intubation. Successful anesthetization was monitored by response to stimuli. No animals met endpoint criteria as defined by: (a) loss of 25% body weight from baseline weight when assigned to the protocol, (b) major organ failure or medical conditions unresponsive to treatment, (c) complete anorexia for 4 days or an inability to feed or drink sufficient nutrients to maintain body weight without assistance for 7 days, (d) distress vocalization unresponsive to treatment or intervention for 7 days, or (e) tumors arising from other than experimental means that grew in excess of 10% of body weight, impaired movement, or ulcerated.

Whole blood was collected into tubes containing EDTA. Plasma was isolated from whole blood via centrifugation, and mononuclear cells were isolated from the blood using Lymphocyte Separation Medium (MP Biomedicals #0850494-CF). Spleen and liver biopsies were collected using a harmonic scalpel and a laparoscope for visualization. The rectum was cleaned of fecal material, and punch biopsies were collected using forceps. Jejunal punch biopsies were acquired using a video-guided endoscopy and biopsy forceps. Biopsies were transported in Roswell Park Memorial Institute media and washed with PBS prior to processing. Mononuclear cells were isolated from tissue biopsies by mashing biopsies through a 0.22 μm cell strainer.

### Flow Cytometry

Flow cytometry was performed on stained mononuclear cells using a Cytek Aurora 5L (Cytek Biosciences, SpectroFlow v3.0.3). Antibodies against the following antigens were used for staining at predetermined concentrations: Ki67 (B56) BUV395, CD4 (OKT4) BUV496, CD3 (SP34-2) BUV563, HLA-DR (G46-6) BUV615, CD450 (DO58-1283) V450, CD20 (2H7) PE, and CD28 (CD28.2) PE-CF594 from BD Biosciences; CD95 (DX2) BV421, CD8 (SK1) BV510, and CD11b (ICRF44) BV570 from BioLegend. Cell viability was assessed using Zombie Green™ Fixable Viability Kit (BioLegend #423111). Cells were permeabilized with the eBioscience Intracellular Fixation & Permeabilization Buffer Set (ThermoFisher Scientific #88-8824-00) prior to intracellular staining. Cell populations were analyzed using FlowJo (FlowJo 10.10.0) and defined as described in Supplementary Figure 1A.

### Fluorescence Assay

Concentrations of Evans blue dye and FITC-Dextran in the plasma were assessed *ex vivo* with a fluorescence assay. A standard curve of FITC-Dextran was prepared at 1 mg/mL in uninfected, untreated plasma and serially diluted 6-fold (1:10). Untreated and uninfected plasma was used as a blank. Samples were collected in a 96-well black plate, in duplicate, and read at excitation 490 nm and emission 520 nm. Concentrations were interpolated from the standard curve.

A standard curve of Evans blue was prepared at 0.1 mg/mL in uninfected, untreated plasma and serially diluted 5-fold (1:5). Untreated and uninfected plasma was used as a blank. Standards and samples were then incubated with an equal volume of Hemoglobind (Biotech Support Group #H0145) for 30 minutes at maximum speed on an orbital shaker, and centrifuged at 10,000 × g for 2 minutes to remove contaminating iron from red blood cells. Supernatants were carefully collected into a 96-well black plate, in duplicate, and read at excitation 540 nm and emission 680 nm. Concentrations were interpolated from the standard curve.

### ELISAs

Concentrations of sCD14 (MyBiosource #MBS740424), iFABP2 (R&D Systems #DC140), and zonulin (ALPCO #30-ZONSHU-E01) were quantified in technical duplicates from plasma using commercially available kits and according to manufacturer’s protocols.

### Viral Loads

Plasma viral RNA levels were determined as previously described [34].

### Albumin

Albumin concentrations were quantified from frozen plasma using the Piccolo Comprehensive Metabolic Panel (Abaxis #400-1028) and a Piccolo blood chemistry analyzer according to manufacturer’s instruction.

### Statistical Analyses

Paired or unpaired *t*-tests and 2-way ANOVAs with multiple comparisons were used, as appropriate, in statistical analyses of derived concentrations and hematopoietic cell fluorescence (GraphPad Prism v10.2.0).

## RESULTS

### Plasma Fluorescence of FITC-Dextran Does Not Reliably Measure Intestinal Permeability in Rhesus Macaques

**Figure 1.**
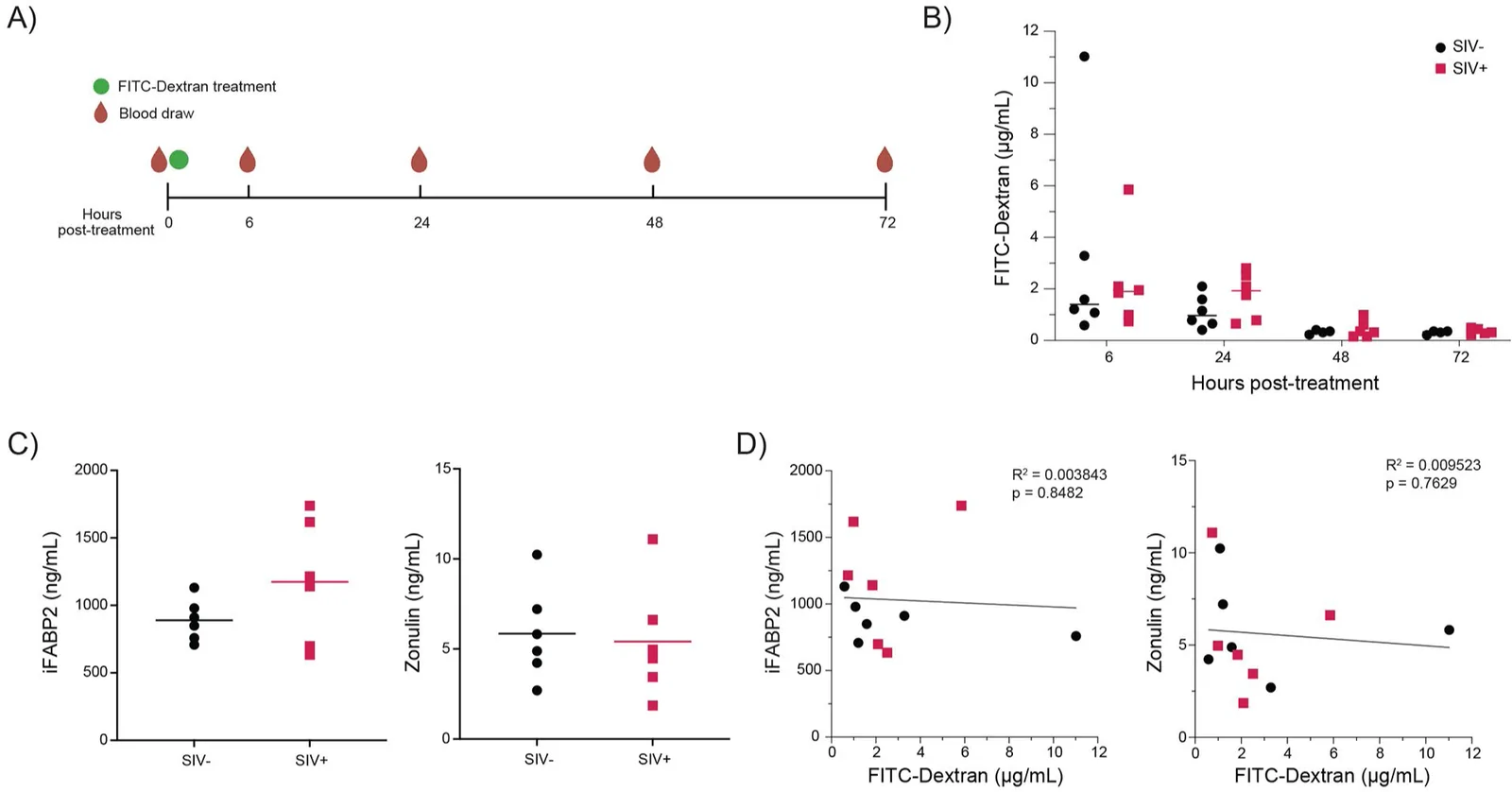
**Systemic measurement of orally administered FITC-Dextran is not a reliable method to measure intestinal dysfunction in nonhuman primates**. A) Study timeline. Animals received a onetime dose of FITC-Dextran, with blood collected prior to treatment and at denoted timepoints following treatment. B) Concentrations of FITC-Dextran calculated using a fluorescence assay. C) Plasma concentrations of iFABP2 and zonulin at the 6-hour post-treatment timepoint. D) Correlations between FITC-Dextran concentrations (fluorescence assay) and markers of intestinal damage iFABP2 and zonulin (ELISA) at the 6-hour post-treatment timepoint. Lines in B and C denote the mean of biological replicates. Trendline in D represents simple linear regression. n = 6 per group as shown.

Fluorescence measurement of plasma following oral administration of FITC-Dextran is an established method of measuring intestinal permeability and microbial translocation in murine models of GI tract damage [35–37]. We sought to determine if FITC-Dextran could be applied to a nonhuman primate model of SIV infection. Six healthy and 6 chronically SIV-infected animals were treated with (500 mg/kg, 4 kDa) FITC-Dextran, and blood was collected at baseline, 6-, 24-, 48-, and 72-hours post-treatment, and fluorescence was measured by spectrofluorometry (Figure 1A). At 6- and 24-hours following treatment, SIV-infected animals had slightly elevated concentrations of FITC-Dextran compared to healthy animals, although not significantly so (Figure 1B). There were similarly no significant differences in iFABP2 or zonulin between the healthy and SIV-infected macaques (Figure 1C) at 6-hours post-treatment and we found no significant correlations between FITC-Dextran concentrations and markers for epithelial damage, iFABP2 and zonulin (Figure 1D). These data do not support FITC-Dextran as an improved or more sensitive method for intestinal permeability in nonhuman primates.

### Evans Blue Can be Detected and Quantified in Rhesus Macaques Following Uracil Treatment or SIV Infection

**Figure 2.**
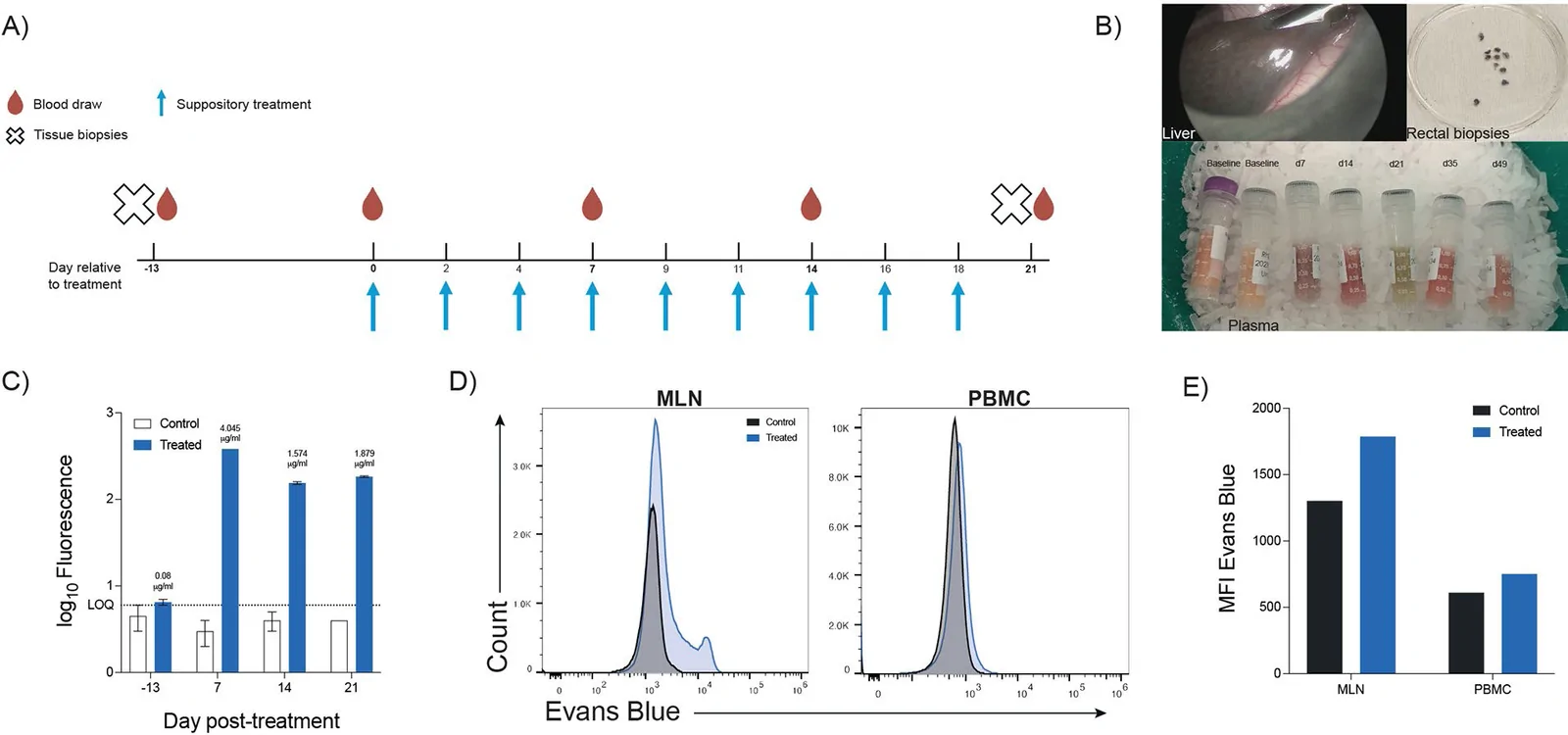
**Suppository-delivered Evans blue can be detected in the plasma and in hematopoietic cells**. A) Representative schedule. Two healthy nonhuman primates were treated intrarectally with either Evans Blue or Normosol-R at the time points indicated by the blue arrows. Blood and tissue biopsies were collected as depicted. B) Images of the liver (taken by endoscopy) and rectal biopsies (*ex vivo*) at day 21 post-treatment, and frozen plasma taken at timepoints relative to treatment initiation as indicated. C) Log_10_ fluorescence of Evans blue in plasma determined by fluorescence assay. The limit of quantification is represented by the dotted line, and interpolated concentrations are written above the bar. D-E) Median fluorescence intensity (MFI) of Evans blue in live hematopoietic (Live CD45^+^) cells from mesenteric lymph node biopsies and peripheral blood mononuclear cells (PBMCs) of each animal at day 21 post-treatment, as measured by flow cytometry and represented by raw histogram (D) bar chart (E). Bar heights in C indicate mean ± SEM of two technical replicates. Bar heights in D indicate raw flow cytometry fluorescence values. n = 1 per group as shown.

Bacterial uracil has been shown to modulate the epithelial barrier in the GI tracts of insects [38, 39]. In an independent investigation to assess the effects of uracil on the gut barrier of nonhuman primates, we aimed to track luminal uracil retention by adding Evans blue to a uracil suppository. Evans blue was chosen because it is a well-characterized inert dye that would not be visually confused with biological specimens [40]. Two healthy macaques received intrarectal treatments 3 times per week with either a cocoa butter suppository containing uracil and Evans blue, or Normosol-R as a control, with sample collections occurring at marked timepoints (Figure 2A). Cocoa butter was selected as the suppository vehicle because the Food and Drug Administration has it listed as an inactive compound, and it is reported to have limited interactions with the intestinal microbiome [41]. To our surprise, the liver and rectal biopsies were visibly blue following 3 weeks of uracil (Evans blue) treatment, and we observed changes in plasma coloration throughout treatment, particularly on day 21 post-treatment (Figure 2B).

Thus, we hypothesized this approach may be a non-invasive methodology to determine colonic permeability. To assess whether Evans blue suppositories might be utilized as a colonic permeability assay, we developed a fluorescence assay and flow cytometry panel to detect and quantify Evans blue in our biological samples. Evans blue was quantifiable in the plasma of the uracil-treated animal throughout treatment, whereas detection in a control animal never surpassed the limit of quantification (Figure 2C). In the treated animal, there was also a distinct increase in median fluorescence intensity (MFI) of Evans blue in hematopoietic cells (Live, CD45^+^) isolated from mesenteric lymph node (MLN) biopsies, as compared to the control animal (Figure 2D and 2E). This shift was not distinct on peripheral blood mononuclear cells (PBMCs, Figure 2D and 2E).

**Figure 3.**
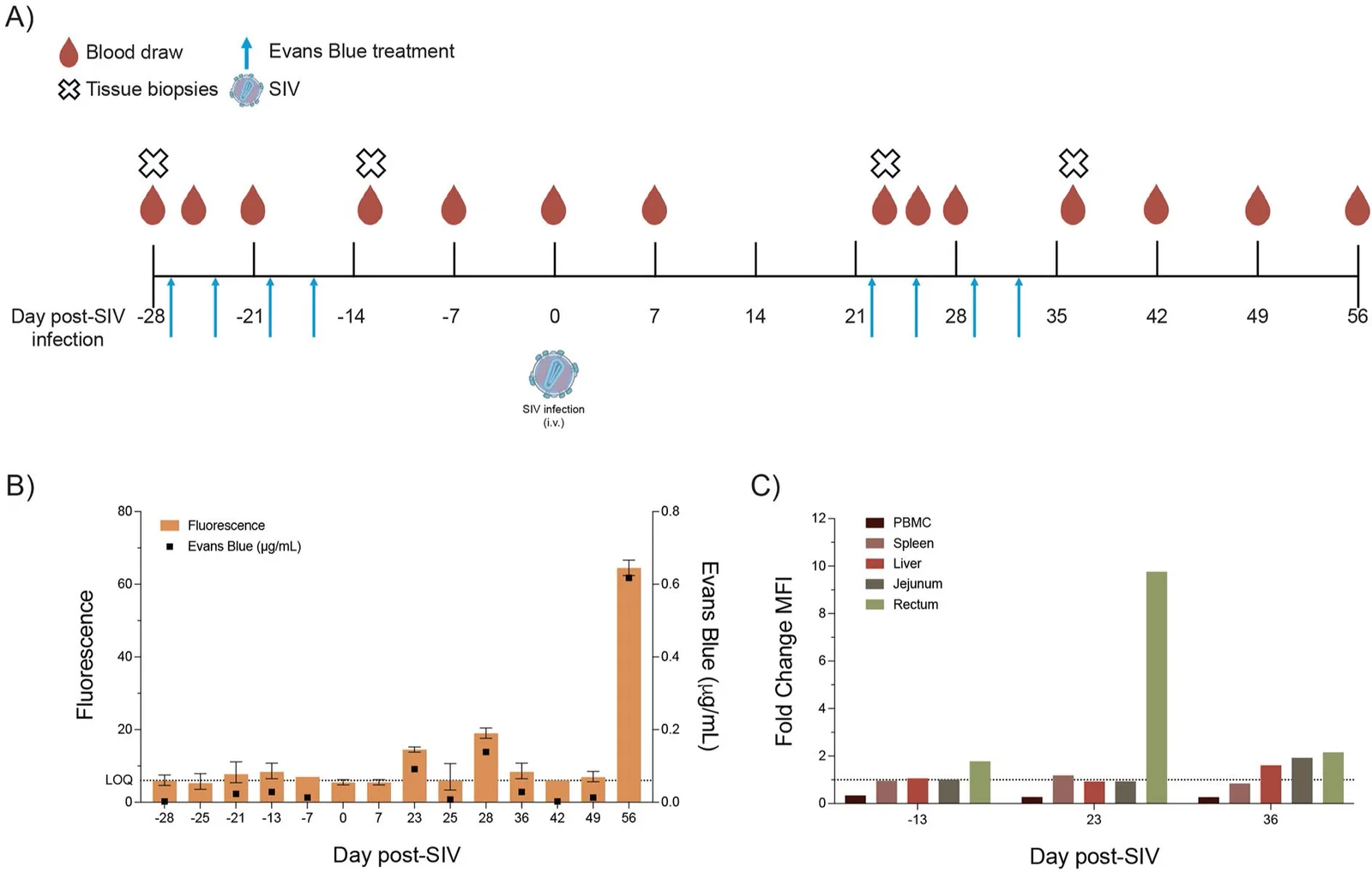
**Evans blue is detectable following acute SIV infection**. A) Representative schedule. Evans blue treatment, denoted by the blue arrows, occurred before and after intravenous SIV infection. Blood and tissue biopsies were collected at depicted timepoints. B) Fluorescence (left axis) and interpolated mean concentrations (right axis) of Evans blue in the plasma. Limit of quantification is represented by the dotted line. C) Fold change in MFI of Evans blue from baseline to depicted timepoint. MFI was determined on live hematopoietic cells from peripheral blood mononuclear cells (PBMCs) and tissue biopsies. Bar heights in B represent mean ± SEM of two technical replicates for the fluorescence and the squares represent the interpolated mean concentration of the fluorescence replicates. Bar heights in C represent 1 normalized biological replicate.

To assess whether Evans blue dissemination could reflect epithelial damage induced by SIV infection, we designed a pilot study in which one animal was intrarectally treated with a short course of Evans blue suppository (without uracil) before and after intravenous SIV infection (Figure 3A). Prior to SIV infection, there was minimal Evans blue detected in the plasma (Figure 3B). Post-SIV infection, Evans blue in the plasma increased, although intermittently, with the maximum concentration occurring on day 56 post-infection at 0.617 μg/mL, more than 3 weeks following the last Evans blue treatment (Figure 3B). We further evaluated Evans blue fluorescence among hematopoietic cells isolated from PBMCs and spleen, liver, jejunal and rectal biopsies. Hematopoietic cells isolated from rectal biopsies manifested the greatest increase (relative to baseline) in MFI of Evans blue at each biopsy timepoint, with the greatest fold change, 9.76, occurring on day 23 post-infection (Figure 3C). By day 36 post-infection, Evans blue detection began to wane in hematopoietic cells from rectal biopsies but marginally increased in liver and jejunal biopsies relative to baseline (to 1.61 and 1.92, respectively; Figure 3C). Taken together, these data support that we can quantify Evans blue in samples collected from nonhuman primates, although its irregular detection following infection may introduce limitations to the method.

### Evans Blue Dye is Not a Reliable Marker of Intestinal Barrier Permeability in Chronically SIV-infected Rhesus Macaques

**Figure 4.**
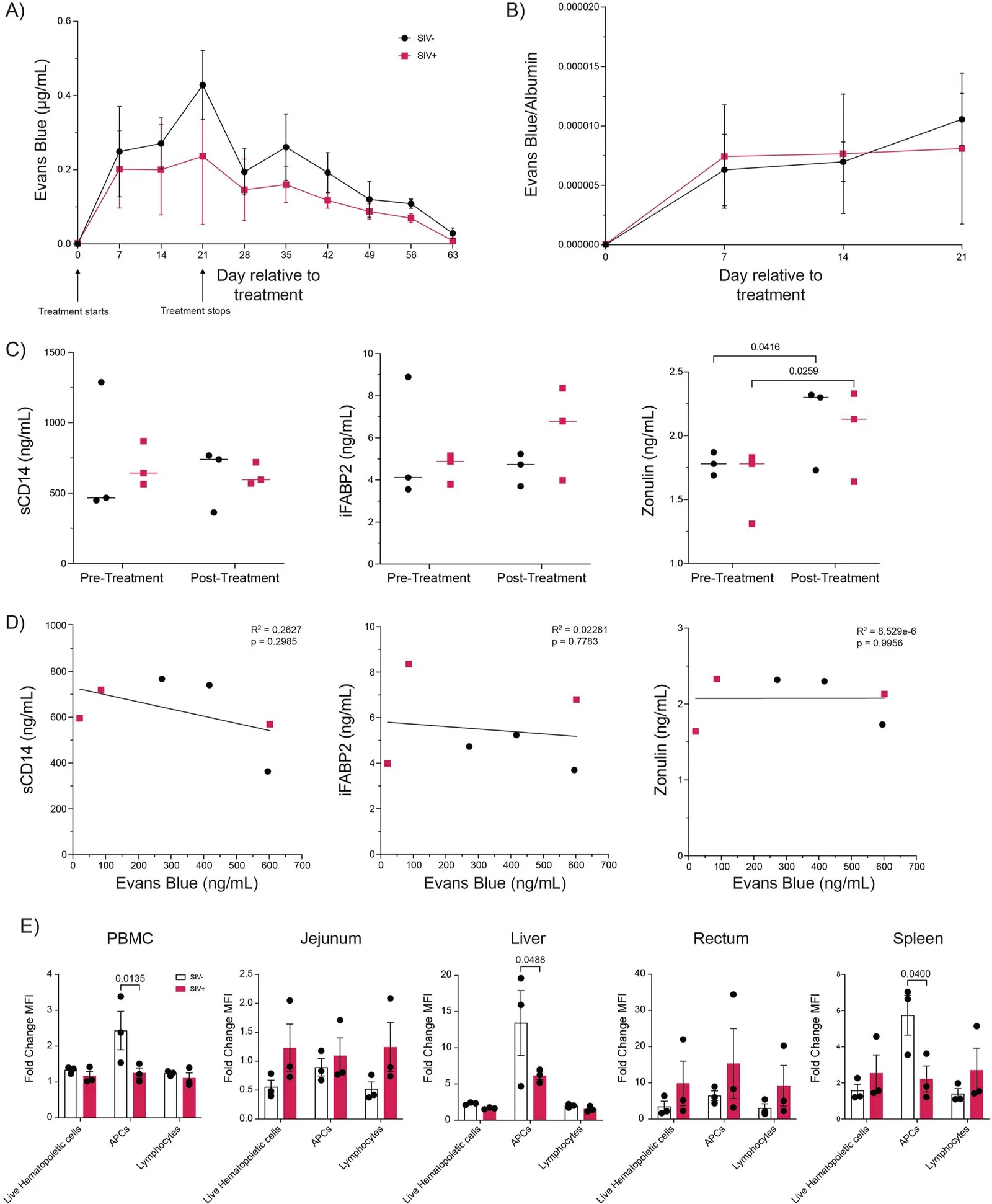
**Evans blue spreads systemically in both SIV-uninfected and chronically SIV-infected animals**. A) Longitudinal concentrations of Evans blue in plasma. B) Ratio of Evans Blue to albumin concentrations during treatment. C) Plasma concentrations of sCD14, iFABP2 and zonulin at day 21 post-treatment. D) Correlations between Evans blue concentrations and markers of intestinal damage sCD14, iFABP2, and zonulin (ELISA), in plasma at day 21 post-treatment. E) Fold change in MFI of Evans blue from baseline to day 21 post treatment in hematopoietic cells isolated from peripheral blood mononuclear cells (PBMCs) and tissue biopsies. Lines in A-B denote mean ± SEM of biological replicates. Lines in C denote the mean of biological replicates. Trendlines in D represent simple linear regression. Bar heights in E denote the mean ± SEM of biological replicates. n = 3 animals per group as shown. Significance in E was assessed by 2-way ANOVA with multiple comparisons.

Progressive SIV infection in nonhuman primates is associated with extensive mucosal barrier damage, increasing rates of microbial translocation [5, 42]. To assess if the suppository-delivered Evans blue assay could be a reliable measurement of damage and translocation, we treated 3 SIV-uninfected and 3 chronically SIV-infected macaques intrarectally with an Evans blue suppository, following the previously established treatment schedule (Figure 2A). To our surprise, on average, healthy animals had higher plasma concentrations of Evans blue compared to the chronically infected animals (Figure 4A). Because Evans blue dye has high affinity towards serum albumin, we assessed albumin concentrations in the plasma throughout the study to determine if the Evans blue trend was due to differences in albumin concentrations [40]. Indeed, albumin differed between SIV-negative and -positive animals (Supplementary Figure 2); however, Evans blue detection did not significantly differ between chronically infected and healthy animals when adjusted for albumin levels (Figure 4B). We also did not observe any significant differences in plasma sCD14, iFABP2, or zonulin by infection status, despite observing that treatment itself led to an unexpected increase in zonulin (Figure 1C). No correlations were evident between plasma concentrations of Evans blue and standard markers of microbial translocation, sCD14, iFABP2, and zonulin, post-treatment (Figure 4D). In hematopoietic cells, fold-changes in MFI (relative to baseline) of Evans blue in both healthy and infected animals were greatest in the rectum and liver (Figure 4E), reflecting our prior visual observations (Figure 2B). Among hematopoietic cells, antigen-presenting cells (APCs) from PBMCs (*P*=0.0135), liver biopsies (*P*=0.0488), and spleen biopsies (*P*=0.0400) of healthy animals had a significantly greater Evans blue detection relative to baseline as compared to infected animals (Figure 4E), with no other differences noted. Overall, these data do not support suppository-delivered Evans blue as a more sensitive and reliable marker for intestinal barrier permeability.

### Uracil Treatment Increases Systemic Evans Blue Dissemination

In our initial experiments using Evans blue as a measurement of suppository uracil retention, we noted a drastic increase of circulating Evans blue in a treated animal despite being SIV-uninfected. This difference in magnitude in response to uracil treatment compared to SIV infection may suggest that uracil is a strong mediator of gut barrier disruption, and thus Evans blue may be an accurate, albeit low-sensitivity assay. For this reason, we further assessed the role of uracil on the GI barrier and its effect on Evans blue uptake.

**Figure 5.**
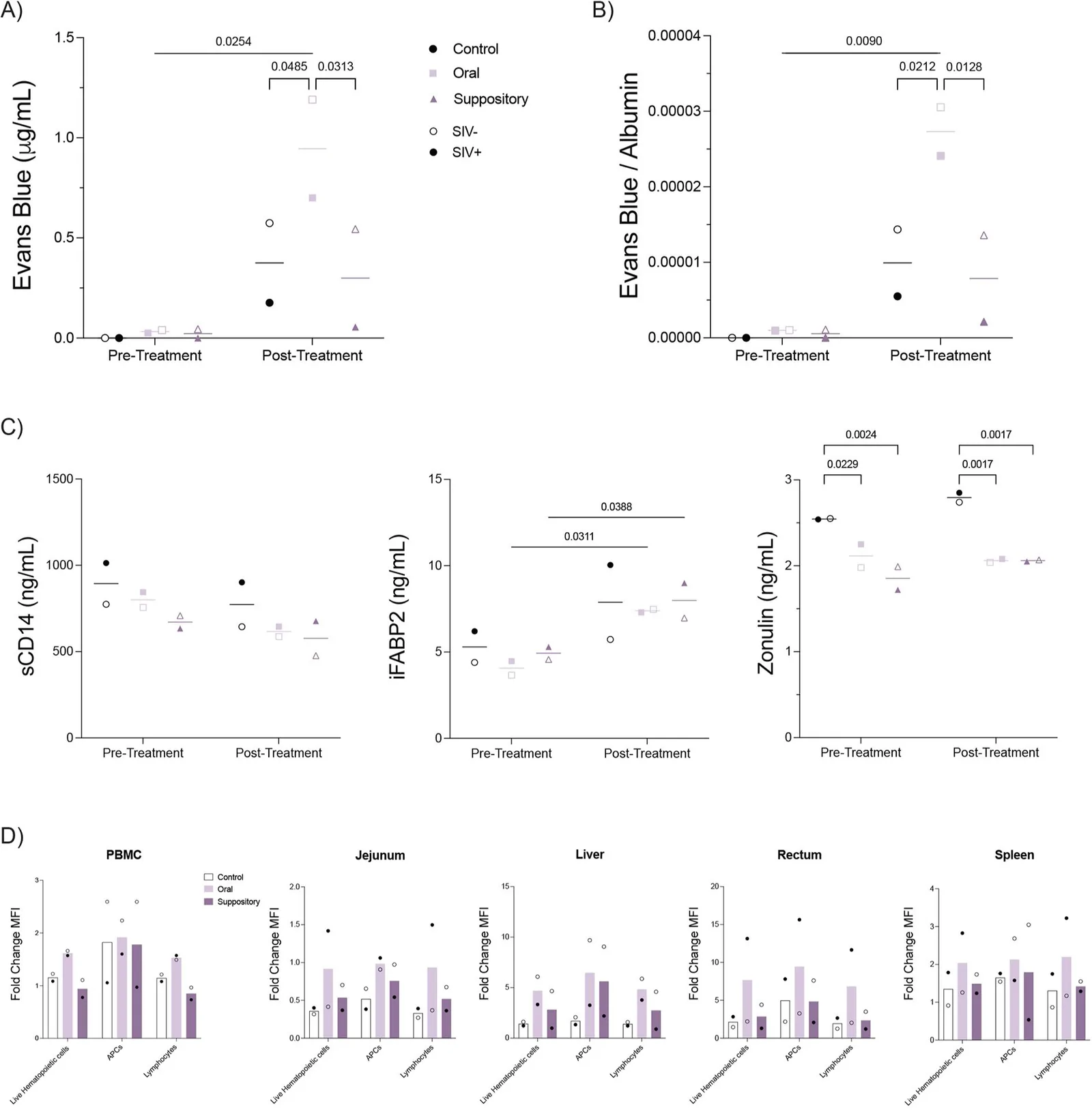
**Uracil modulates gut barrier integrity**. A) Plasma concentrations of Evans blue pre- and post-treatment were assessed by fluorescence assay. B) Ratio of Evans blue to albumin before and after treatment. C) Concentrations of intestinal damage markers sCD14, iFABP2, and zonulin were assessed from plasma using ELISAs. D) Fold change in MFI of Evans blue from pre- to post-treatment on hematopoietic cells from peripheral blood mononuclear cells (PBMCs) and tissue biopsies collected post-treatment. Open shapes represent SIV-uninfected animals, closed shapes represent SIV-infected animals. Lines and bar heights in A-D denote the mean ± SEM of biological replicates. n = 2 per group as shown. Significance in A-D was assessed by 2-way ANOVA with multiple comparisons. P-value lines without brackets represent within group longitudinal comparisons, and lines with brackets represent between group comparisons.

Following the same 3-times-weekly treatment and sampling schedule previously established (Figure 2A), 3 healthy and 3 chronically infected animals were divided into oral uracil, suppository uracil, or control groups (no uracil), with 1 healthy and 1 infected animal per group. All animals received intrarectal Evans blue treatments – animals in the suppository group had uracil added to the cocoa butter, those in the oral group were provided daily doses of uracil in their food, and animals in the control groups had no addition of uracil. Animals receiving oral uracil had significantly greater concentrations of Evans blue in the plasma compared to baseline (*P*=0.0254), as well as compared to the suppository (*P*=0.0313) and control (*P*=0.0485) groups post-treatment (Figure 5A). We continued to observe that SIV-uninfected animals had higher concentrations of Evans blue in all treatment groups (Figure 5A). When normalized to albumin levels, the oral uracil group still had significantly more Evans blue compared to baseline (*P*=0.009), as well as compared to the suppository (*P*=0.0128) and control (*P*=0.0212) groups post-treatment – albumin adjustment did not alter infection status rank-order (Figure 5B) as previously observed in Figure 4.

When assessing concentrations of plasma sCD14, we did not see any differences between groups or following treatment (Figure 5C). However, we did find significant longitudinal increases in iFABP2 in oral (*P*=0.0311) and suppository (*P*=0.0388) groups following treatment (Figure 5C). These changes were not evident in the control group nor between groups post-treatment (Figure 5C). Interestingly, plasma zonulin concentrations in the control group were significantly higher than in the oral and suppository groups both pre- (*P*=0.0229, *P*=0.0024) and post-treatment (*P*=0.0017), although no differences were noted longitudinally (Figure 5C). We again assessed Evans blue in all hematopoietic cells, APCs, and lymphocytes across tissue sites. There were no significant differences in MFI fold-changes (from baseline) of Evans blue between the groups, although uracil treatment, regardless of route, trended towards greater increased detection compared to control, particularly in the liver and rectum (Figure 5D). Our data suggest that uracil treatment, particularly oral uracil, may influence epithelial barrier integrity in nonhuman primates. However, taken together, our results indicate that suppository-based Evans blue is neither a reliable nor improved method for assessing epithelial damage or microbial translocation in SIV infection in nonhuman primates.

## DISCUSSION

Microbial translocation is a key driver of chronic immune activation during SIV infection, contributing to disease progression and the development of non-AIDS comorbidities [7]. Efforts to understand the impact and mechanisms of microbial translocation provide insight into disease pathogenesis and may reveal novel therapeutic targets. Our methodological study aimed to develop an improved and more direct method for measuring microbial translocation through use of tracers FITC-Dextran and Evans blue dye to assess intestinal barrier permeability during SIV infection in rhesus macaques. Our results indicate that use of these tracers does not provide an improved or reliable method for quantifying intestinal barrier permeability. Indeed, treatment with compounded Evans blue led to an unexpected increase in circulating zonulin concentrations. Further understanding of the mechanisms underlying microbial translocation would be beneficial for the effective development of more accurate and reliable methods.

Limited studies have applied *in vivo* administration of FITC-Dextran to explore intestinal barrier disruption in nonhuman primate models. Similar to our results, a study aiming to understand barrier disruption in a nonhuman primate model of Parkinson’s disease did not report increased FITC-Dextran dissemination in Parkinson’s disease-induced animals [30]. This highlights that the limitations of FITC-Dextran may reflect distinct GI processes in nonhuman primates compared to humans and murine models. In Cynomolgus macaques, the gastric half-emptying rate was reported as between 21 and 27 minutes, similar to what is reported in humans, and the oral-cecal transit time was between 2.3 and 2.5 hours, an average of 1.5 hours shorter than in humans, and significantly longer than what has been reported in mice [36, 43]. It may be reasonable to assume that, in our pilot study exploring the application of FITC-Dextran to SIV infection, our first sampling time point at 6 hours had surpassed the peak of absorption, and further optimization of this method may reveal improved results.

While we observed an unreliable increase in Evans blue detection during acute SIV infection (Figure 3), we did not observe the expected trend in chronically infected animals (Figure 4). Azo dyes, like Evans blue, can be toxic, causing acute inflammation or even death at high doses in animal models [40, 44]. In rhesus macaques, an intravenous dose of Evans blue dye more than 50 mg/kg resulted in death within 11 days, whereas an intravenous dose of 25 mg/kg was found to be nontoxic [44]. While our treatments equated to more than 50 mg/kg per dose, these were delivered intrarectally as opposed to intravenously, and as observed (Figure 3, 4, and 5), Evans blue did not enter the bloodstream at this concentration. We did not assess whether Evans blue or cocoa butter alone contributed to the increase in intestinal permeability observed with the compounded formulation (Figure 4).

Immunological dysfunction in macrophages resulting from SIV infection may influence our study results at distal sites and provide a reasonable explanation for the differences we observe in Evans blue detection between healthy and chronically infected animals [45–47]. In the spleen, SIV infection influences myeloid cell differentiation to promote healing rather than an inflammatory response [46]. Splenic macrophages from SIV-infected animals are functionally distinct from healthy counterparts, specifically, in that their responsiveness to LPS is limited to an expansion of IL-6 monofunctional cells [47]. IL-6 is pleiotropic and has both pro- and anti-inflammatory functions on macrophages [48]. In the liver, the accumulation of microbial products contributes to long-term damage, which is marked by an increase in absolute Kupffer cell numbers, apoptosis, and turnover [45, 49]. Increased turnover of Kupffer cells could influence the frequency and presence of mature, fully functional cells, which in turn may result in decreased phagocytic activity within the liver [45]. Thus, altered phagocytic activity during SIV infection may influence Evans blue detection within APCs.

Albumin production can be impaired as a result of SIV-induced liver failure, which may contribute to our inability to reliably use Evans blue concentrations in the plasma to approximate intestinal dysfunction [50]. Although we confirmed that albumin production was indeed impaired in chronically infected animals (Supplementary Figure 2), normalizing Evans blue concentrations to the amount of albumin present only modestly adjusted the observed trends in detectable Evans blue (Figure 4). Moreover, our group has established that microbial translocation is not stochastic, but rather biased, perhaps suggesting that translocation is not a passive process [16, 51]. This highlights an opportunity to further investigate the mechanisms underlying preferential translocation, and how that may play a role in tracer translocation.

In *Drosophila*, bacterial-derived uracil induces intestinal reactive oxygen species production in a dual oxidase (DUOX)-dependent manner [38]. Our group has recently established that in otherwise healthy animals, exogenous oral uracil treatment increases susceptibility to SIV independently of intestinal inflammation [52]. Here, our results further demonstrate that although both oral and suppository uracil modulate epithelial integrity, only oral uracil results in an increase in Evans blue in the plasma. Interestingly, oral uracil induced greater Evans blue accumulation within rectal hematopoietic cells compared to suppository uracil and control groups, suggesting that oral uracil is a modest inducer of intestinal epithelial damage. Curiously, however, we see that oral uracil has a greater effect on healthy animals rather than chronically infected animals. The reasons for this are unclear but, may suggest that uracil amplifies a mechanism of Evans blue transport that is otherwise repressed during chronic SIV infection. A deeper understanding of the mechanisms of macromolecular transport across the intestinal epithelium will inform both our results and our understanding of the causes and consequences of microbial translocation overall.

In summary, we assessed the application of 2 tracers, FITC-Dextran and Evans blue, as methods for measuring intestinal barrier permeability. Our findings do not support either method as a means of measuring intestinal barrier permeability in non-human primates. Although the small number of animals in our study precluded detection of increased post-infection intestinal permeability using traditional non-invasive methods, we conclude that Evans blue did not improve sensitivity. Overall, there is a need for further development of modalities to noninvasively approximate GI tract dysfunction. Despite their limitations, circulating biomarkers currently remain among the most practical and widely used surrogate measures [20, 21].

## SUPPLEMENTARY DATA

Supplementary materials are available at the *Pathogens and Immunity* website. Supplementary data may be provided by the authors to benefit the reader. Supplementary data are not copyedited and are the sole responsibility of the authors. Questions or comments related to supplementary materials should be addressed to the corresponding author.
