# Supplementary material for: Systemic Evaluation of Administered FITC-Dextran or Evans Blue Dye to Determine Intestinal Barrier Permeability in Nonhuman Primates
Source: Pathog Immun. 2026 Aug 25;11(2):96–115. doi: 10.20411/pai.v11i2.1054 (PMC13521241; doi:10.20411/pai.v11i2.1054)
Supplement: Supplementary file 1 [file pai-11-096-s01.pdf]

PATHOGENS AND IMMUNITY
IN ASSOCIATION WITH CASE WESTERN RESERVE UNIVERSITY AND THE RICHARD J. FASENMYER FOUNDATION

Supplementary Figure 1

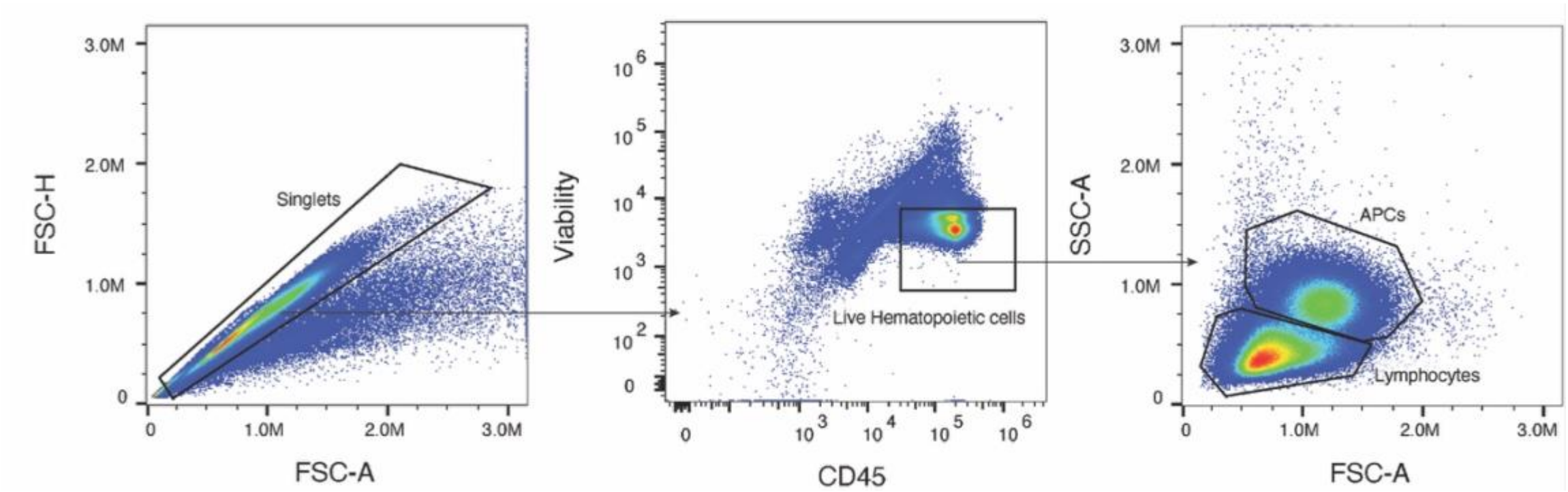

Supplementary Figure 1. Representative gating scheme. Cells gated as singlets were characterized as live (Zombie Green$^-$) hematopoietic (CD45$^+$) cells before being classified as lymphocytes or antigen presenting cells (APCs) via size exclusion.

Bodykevich EG, Vinton CL, Ortiz AM, Brenchley JM. Systemic Evaluation of Administered FITC-Dextran or Evans Blue Dye to Determine Intestinal Barrier Permeability in Nonhuman Primates. *Pathogens and Immunity*. 2026;11(2):96–115. doi: 10.20411/pai.v11i2.1054

PATHOGENS AND IMMUNITY
In Association with Case Western Reserve University and The Richard J. Fasenmyer Foundation

Supplementary Figure 2

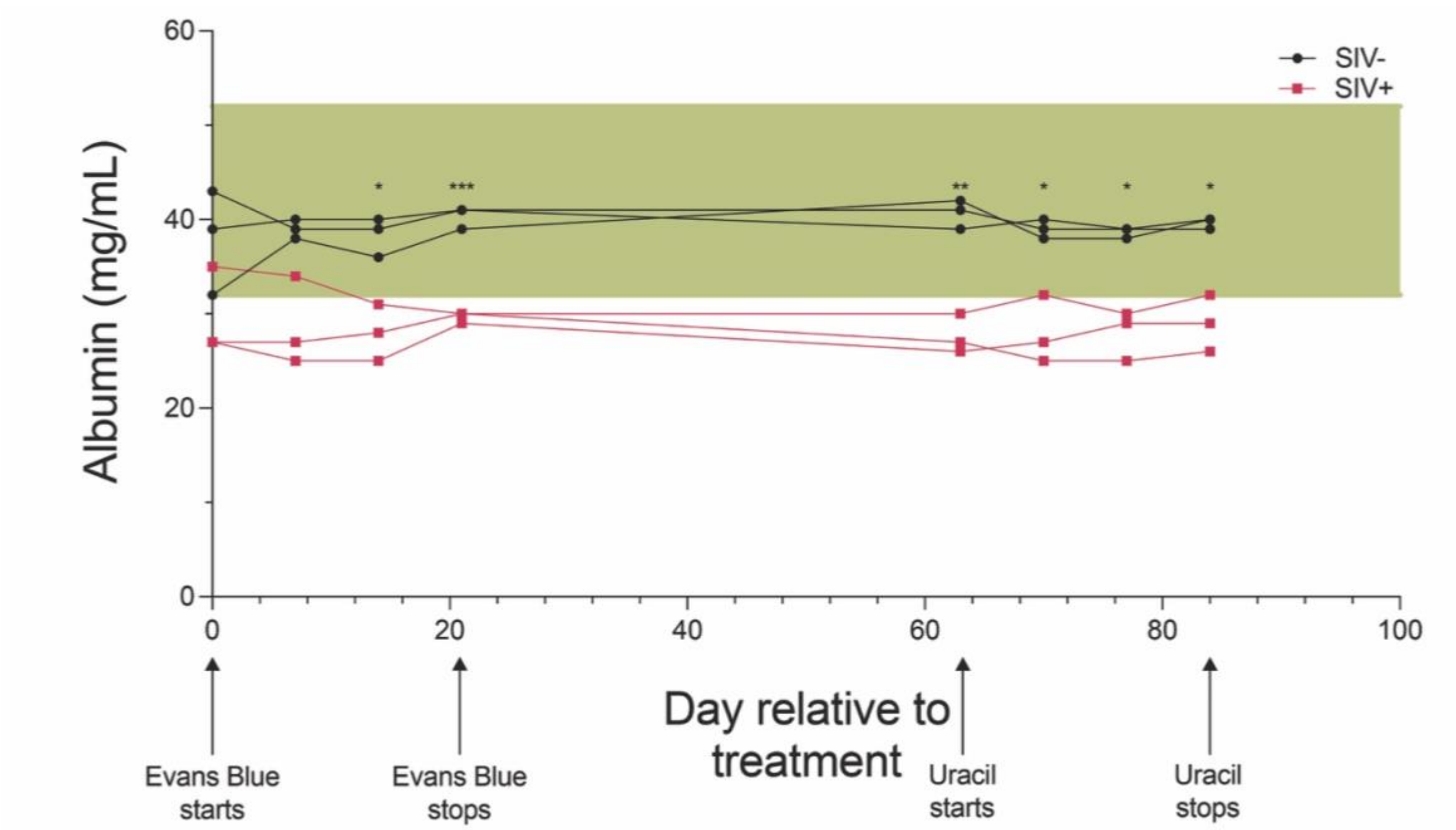

Supplementary Figure 2. Albumin levels in study animals. Albumin concentrations in healthy and chronically SIV-infected animals. The area shaded green represents the reference range for serum albumin in nonhuman primates. Significance was assessed by 2-way ANOVA with multiple comparisons.

PATHOGENS AND IMMUNITY
IN ASSOCIATION WITH CASE WESTERN RESERVE UNIVERSITY AND THE RICHARD J. FASENMYER FOUNDATION

Supplementary Table 1

Animal	Day post-SIV infection	Viral loads (SIVgag/mL)	Treatment group	Genotype
RM1	669 (3000 $TCID_{50}$ intravenously)	1,400,000	FITC-Dextran	A01+B08-B17-A02-
RM2	455 (4 $TCID_{50}$, intrarectally)	1,100,000	FITC-Dextran	A01+B08-B17+
RM3	596 (4 $TCID_{50}$, intrarectally)	1,500,000	FITC-Dextran	A01+B08-B17-A02-
RM4	690 (3000 $TCID_{50}$ intravenously)	1,600,000	FITC-Dextran	A01-B08-B17-
RM5	305 (3000 $TCID_{50}$ intravenously)	1,200,000	FITC-Dextran	A01-B08-B17-A02-
RM6	Uninfected	N/A	FITC-Dextran	A01+B08-B17+A02-
RM7	Uninfected	N/A	FITC-Dextran	A01+B08-B17-A02-
RM8	Uninfected	N/A	FITC-Dextran	A01+B08-B17-A02-
RM9	Uninfected	N/A	FITC-Dextran	A01+B08-B17+A02-
RM10	Uninfected	N/A	FITC-Dextran	A01-B08-B17+A02-
RM11	Uninfected	N/A	FITC-Dextran	A01+B08-B17-A02-
RM12	Uninfected	N/A	Evans Blue + suppository uracil	A01-B08-B17-A02-
RM13	Uninfected (Figure 2); 751 (Figure 4 and 5) (4 $TCID_{50}$, intrarectally)	1,500,000 (Figure 4 and 5)	Control; Evans Blue + suppository uracil	A01-B08-B17-
RM14	Uninfected, infected intravenously during experiment (1000 $TCID_{50}$)	N/A	Evans Blue	A01-B08-B17-
RM15	Uninfected	N/A	Evans Blue + suppository uracil	A01-B08-B17-
RM16	Uninfected	N/A	Evans Blue + oral uracil	A01-B08-B17+
RM17	394 (4 $TCID_{50}$, intrarectally)	680	Evans Blue + oral uracil	A01+B08-B17+A02-
RM18	Uninfected	N/A	Control	A01+B08-B17-
RM19	364 (4 $TCID_{50}$, intrarectally)	1,600,000	Control	A01+B08-B17-A02-

Bodykevich EG, Vinton CL, Ortiz AM, Brenchley JM. Systemic Evaluation of Administered FITC-Dextran or Evans Blue Dye to Determine Intestinal Barrier Permeability in Nonhuman Primates. *Pathogens and Immunity*. 2026;11(2):96–115. doi: 10.20411/pai.v11i2.1054

PATHOGENS AND IMMUNITY
IN ASSOCIATION WITH CASE WESTERN RESERVE UNIVERSITY AND THE RICHARD J. FASENMYER FOUNDATION

Supplementary Table 1. Study animals. A total of 19 animals were used in these studies. Infection status at the start of experiment, treatment group, SIV viral loads at study initiation, and MHC genotypes are included. RM13 was used as a healthy negative control in the development of the protocols (Figure 2). It was infected outside the scope of this study and was used as a chronically infected animal before (Figure 4) and after (Figure 5) receiving suppository uracil. RM14 was used in the pilot study (Figure 3) and was SIV-infected intravenously during this study.
